# Mecanismos para el fortalecimiento de la investigación clínica: la experiencia colombiana

**DOI:** 10.26633/RPSP.2019.70

**Published:** 2019-08-22

**Authors:** Laura Angélica Pineda Velandia, Francisco Javier Sierra Esteban

**Affiliations:** 1 Investigadores independientes Investigadores independientes Bogotá Colombia Investigadores independientes, Bogotá, Colombia.

**Keywords:** Ensayos clínicos como asunto, regulación gubernamental, Colombia, Clinical trials as topic, government regulation, Colombia., Ensaios clínicos como assunto, regulamentação governamental, Colômbia.

## Abstract

En este artículo se describen los cambios regulatorios y los procesos implementados en Colombia que permitieron crear las condiciones técnicas favorables para la investigación clínica con medicamentos en el país. El impacto de las medidas tomadas se midió en términos del número de centros de investigación certificados en buenas prácticas clínicas, los tiempos de evaluación de los protocolos de estudios clínicos y la calidad de los conceptos emitidos. Mediante el uso de fuentes de información públicas o datos de la literatura, se pudo determinar que el establecimiento de la regulación que exige la certificación en buenas prácticas clínicas y el cambio de procedimiento de evaluación de los protocolos de estudios clínicos contribuyeron a la calidad y oportunidad de la investigación clínica en Colombia. A pesar de los resultados obtenidos, es claro que, además del fortalecimiento de las agencias regulatorias, se debe llevar a cabo una revisión y actualización de la regulación relacionada con otros actores del ecosistema de investigación clínica para que se puedan garantizar condiciones seguras para la realización de estudios clínicos junto con un incremento del número de estudios que se efectúan en el país.

## Abstract (en)

This article describes the regulatory changes and procedures introduced in Colombia that created favorable technical conditions for clinical trials of drugs in the country. The impact of these measures was measured in terms of the number of research centers certified in good clinical practice, the time taken to evaluate clinical trial protocols, and the quality of the concepts developed. Using public information sources or data from the literature, the study found that adoption of the regulation requiring certification in good clinical practice and the change in the procedure for evaluating clinical trial protocols contributed to the quality and timeliness of clinical research in Colombia. Nevertheless, it is clear that the strengthening of regulatory agencies should be accompanied by the review and amendment of the regulations governing other actors in the clinical research ecosystem to guarantee the safety of clinical trials and that more studies should be conducted in the country.

## RESUMO

Neste artigo é descrita a reforma regulatória e os processos implementados na Colômbia que possibilitaram criar condições técnicas favoráveis para a pesquisa clínica com medicamentos no país. A repercussão das medidas adotadas foi avaliada com base no número de centros de pesquisa certificados em boas práticas clínicas, prazos para análise dos protocolos de estudos clínicos e qualidade dos pareceres emitidos. Com a consulta a fontes públicas de informação ou dados da literatura, verificou-se que a instauração de regulamentação para certificação em boas práticas clínicas e a mudança do processo de análise dos protocolos de estudos clínicos contribuíram para a qualidade e a condução oportuna da pesquisa clínica na Colômbia. Apesar dos resultados obtidos, é evidente que, além do fortalecimento dos órgãos reguladores, faz-se necessário examinar e atualizar a regulamentação relacionada a outros atores do ecossistema de pesquisa clínica para garantir condições seguras para a condução de estudos clínicos e o aumento do volume de estudos realizados no país.

El desarrollo de lineamientos técnicos y éticos necesarios para la conducción de estudios clínicos en el mundo ha tenido una evolución constante para garantizar la protección de los sujetos participantes y lograr resultados confiables que permitan el desarrollo de tecnologías en salud nuevas, seguras y eficaces (1). Esto, sin duda, ha influido en el incremento del número de estudios clínicos desarrollados y en la identificación de la investigación clínica como una actividad que genera desarrollo científico y, al mismo tiempo, puede ser una fuente de generación de empleo e ingresos para la economía de los países (2).

En este contexto, el objetivo de este documento es presentar la relación entre los desarrollos regulatorios y procedimentales en investigación clínica y su impacto en los centros de investigación, tiempos de evaluación y calidad de los conceptos de evaluación.

## Desarrollo normativo de la investigación clínica en Colombia

En 1993, el Ministerio de Salud establece los requisitos para la investigación en salud en Colombia. Cuatro años más tarde, la responsabilidad de la evaluación de protocolos de investigación en medicamentos en seres humanos se traslada al Instituto Nacional de Vigilancia de Medicamentos y Alimentos (INVIMA) (3, 4). En 2008, mediante la Resolución 2378, Colombia adoptó las Buenas Prácticas Clínicas (BPC) como el “estándar para el diseño, conducción, realización, monitoreo, auditoría, registro, análisis y reporte de estudios clínicos que proporciona una garantía de que los datos y los resultados reportados son creíbles y precisos y de que están protegidos los derechos, integridad y confidencialidad de los sujetos del estudio” con la observancia de las recomendaciones de la Red Panamericana de Armonización de la Reglamentación Farmacéutica (Red PARF) de 2005 (5). La Resolución 2378 establece, entre otras cosas, las condiciones para la implementación y verificación de las BPC por parte del INVIMA y ratifica la exigencia de aprobación de estudios clínicos por este ente regulatorio (6). El cambio normativo más reciente fue el traslado de la evaluación de estudios clínicos desde la sala especializada de medicamentos al grupo de investigación clínica (GIC) de la Dirección de medicamentos y productos biológicos (7).

**FIGURA 1.**
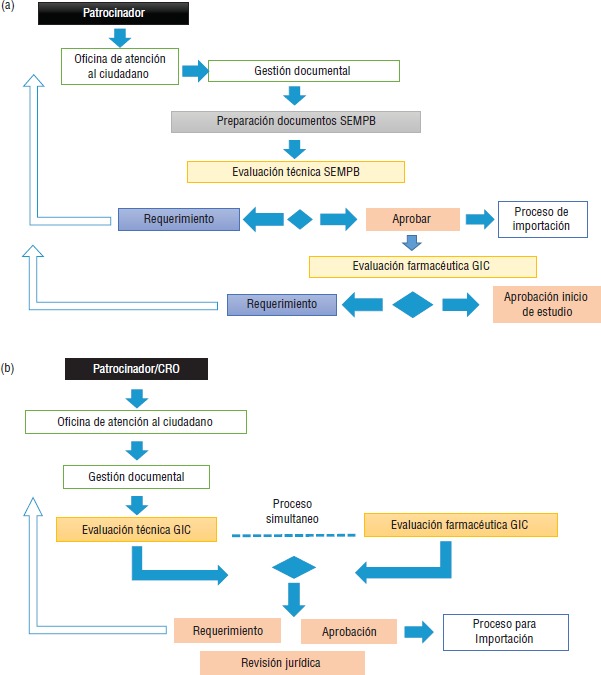
Procedimiento de evaluación de protocolos de investigación. A, antes del Acuerdo 003 de 2016; B, después de dicho acuerdo

***Fuente***: elaboración propia a partir de Acuerdo 003 de 2016 (7).

SEMPB, sala especializada de medicamentos y productos biológicos; GIC, grupo de investigación clínica; CRO, organización de investigación por contrato (por sus siglas en inglés).

## Centros de investigación clínica

Con la expedición de la norma y considerando el período de transitoriedad otorgado para su cumplimiento, a partir de 2010 y hasta 2013 se presentó un incremento sostenido del número de centros certificados por el INVIMA para investigación clínica, que pasaron de dos a 106 centros en ese período. A partir del 2014, el incremento ha sido menor y se llegó a 119 centros en 2018. El aumento moderado de centros de investigación certificados desde la aplicación de la norma se puede explicar, entre otras razones, por una mayor rigurosidad de las exigencias técnicas contenidas en las BPC adoptadas y los retos que enfrentan los centros de investigación en el cumplimento permanente de los requisitos que aseguran el buen desarrollo de los estudios clínicos y la seguridad de los participantes (1). Además de la entrada en vigencia de la norma, la capacidad operativa de los centros para su cumplimiento, y su interés por la investigación, se desconoce si otros factores, tales como incentivos económicos, también podrían explicar el crecimiento del número de centros de investigación desde 2010.

## Procedimiento de evaluación de estudios clínicos y número de protocolos de investigación clínica

En junio de 2016, el INVIMA modifica el procedimiento de evaluación de los estudios clínicos para mejorar la eficacia de la oportunidad de respuesta sin comprometer la calidad de la evaluación. El principal cambio introducido fue trasladar la evaluación de los estudios clínicos de la sala especializada de medicamentos al grupo de investigación clínica (GIC). Con este cambio, se logró eliminar los tiempos administrativos relacionados con el agendamiento de los trámites a la sala especializada y la evaluación simultánea de los aspectos de eficacia, seguridad y calidad de los estudios clínicos y productos en investigación (figura 1).

En relación con el número total de estudios clínicos presentados ante el INVIMA entre 2014 y 2018, el total por año varía entre 85 y 90 (8). Al comparar la tasa de estudios realizados en países de América Latina según su tamaño poblacional en 2018, Chile, Argentina, Brasil y Perú ocupan los primeros lugares con una tasa entre 8 y 3 estudios por 100 000 mil habitantes. Colombia, con una tasa de 2,4 estudios, iguala a México en este indicador (9, 10).

## Tiempo de evaluación de protocolos

**FIGURA 2.**
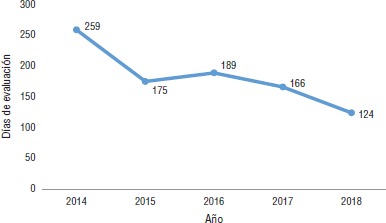
Número de días empleados en la evaluación (desde la radicación hasta el concepto final) de protocolos de estudios clínicos

Con respecto a 2014, en 2018, y como producto de los ajustes incluidos en el proceso, se puede observar una disminución de alrededor de 50% en el tiempo de evaluación de los protocolos de investigación clínica sometidos al INVIMA, que pasó de 259 a 124 días (de ocho a cuatro meses, aproximadamente) (figura 2). Este resultado se debe a que, con el procedimiento anterior, el tiempo para la evaluación por la sala especializada era de aproximadamente 4,5 meses, en los que no se incluía el tiempo para la evaluación farmacéutica y de requerimientos (cuatro meses adicionales) antes del concepto final. Con el procedimiento actual, la evaluación inicial que incluye la revisión técnica y farmacéutica demora un promedio de dos meses y suele finalizar dos meses después con la evaluación de requerimientos y emisión de concepto final.

Al relacionar la información de las tasas del número de estudios por 100 000 habitantes (9, 10) con los tiempos de evaluación de los estudios clínicos de las agencias regulatorias publicados en la literatura (11), no se observa una asociación positiva. Esto se refleja en los datos de países como Argentina y Brasil, con tasas de estudios por 100 000 habitantes de 5,6 y 3,1, respectivamente (9, 10), pero con tiempos de evaluación de aproximadamente nueve meses. Por otra parte, la tasa de estudios de Colombia fue de solo 2,4 estudios por 100 000 habitantes y tiempos de evaluación de cuatro meses. Este análisis permite plantear la hipótesis de que el tiempo de evaluación de los protocolos de investigación clínica por parte de las agencias regulatorias no es la única razón que determina la cantidad de estudios que se realizan en un país. En este contexto, otros factores como la concordancia de perfiles epidemiológicos de los países y las especialidades de los estudios clínicos propuestos a nivel global; las características de factibilidad técnica y económica de los centros de investigación; el potencial de reclutamiento de participantes; la capacidad de respuesta y cumplimiento de estándares de calidad de los comités de ética (1); y la disponibilidad de recurso humano, entre otros, podrían incluirse como otros eslabones del proceso de investigación clínica que explicarían la variabilidad del número de estudios de investigación clínica sometidos a evaluación ante la agencia regulatoria.

***Fuente***: elaboración propia a partir de la base de datos publicada por el INVIMA (8).

## Concepto y calidad de la evaluación

Cuando se revisa el total de estudios con concepto final de evaluación cada año, esto es, una vez que los interesados responden a los requerimientos de información adicional o preguntas de los evaluadores, se observa que el porcentaje de aprobaciones está entre 80% y 95%, mientras que el porcentaje de estudios negados ha sido de aproximadamente 5%. Además de los estudios desistidos por los interesados, el total de conceptos restantes corresponde a los estudios que fueron requeridos y no alcanzaron a recibir concepto final durante el mismo año y su decisión final queda registrada en el año siguiente.

Con respecto a la calidad de la evaluación, cualquiera sea el concepto final de aprobación o negación emitido por la agencia, desde el segundo semestre de 2016 y hasta 2018 se ha evidenciado que un mayor número de estudios evaluados cada año reciben requerimientos técnicos y desistimientos por parte de los interesados (cerca de 80% y 7%, respectivamente). Esto se explica al considerar que el nuevo procedimiento implementado permite una revisión más detallada de los protocolos por parte de un grupo técnico que se dedica de forma exclusiva a esta actividad. Así, la decisión sobre el estudio se soporta en un proceso reproducible y conceptos finales más robustos e integrales.

Entre los temas requeridos, se incluyen aspectos clínicos del estudio (por ejemplo, aclaraciones sobre las preguntas y poblaciones de estudio, antecedentes clínicos y preclínicos de toxicidad y seguridad), la calidad del producto en investigación (información de estabilidad fisicoquímica del medicamento, condiciones de almacenamiento y ajuste de información en etiquetas del producto en investigación), las condiciones éticas y operativas para su desarrollo (por ejemplo, por ajustes en el lenguaje de consentimiento y asentimiento informado, información aclaratoria sobre protección de datos de sujetos y cubrimiento de las pólizas) y documentación administrativa de soporte, principalmente.

## CONCLUSIONES

En los últimos diez años, Colombia ha logrado avanzar en la implementación de las buenas prácticas clínicas para el desarrollo de investigación clínica con medicamentos en seres humanos orientado a la protección de los sujetos participantes y la calidad de los datos obtenidos. Los avances regulatorios en esta materia han permitido crear condiciones técnicas apropiadas para el desarrollo de ensayos clínicos con medicamentos, ya que se dispone de centros calificados para la investigación y de procesos ágiles y robustos para la evaluación de los ensayos clínicos. Con los cambios procedimentales implementados por el INVIMA en 2016, se ha logrado promover el desarrollo de capacidades técnicas y conocimientos de los profesionales responsables de la evaluación de los protocolos de investigación en Colombia, mayor profundidad y calidad en las evaluaciones de los estudios clínicos y optimización del proceso en términos de reducción del tiempo de evaluación. Tener procesos de evaluación que combinan calidad, reproducibilidad y oportunidad puede redundar en un incremento del número de estudios clínicos que se desarrollan en el país, lo cual debe medirse en forma sistemática en el mediano y largo plazo.

Si bien los cambios normativos y procedimentales implementados por la agencia regulatoria son algunas de las acciones necesarias para mejorar la calidad y oportunidad de la evaluación de protocolos de investigación clínica, es necesario explorar y abordar otros factores y actores determinantes para contar con un mayor número de estudios clínicos en el país, como se sugirió a partir del análisis de indicadores en la Región. Entre estos aspectos, el seguimiento al funcionamiento y calidad de los comités de ética en investigación es quizás uno de los de mayor importancia.

Por último, si bien el país ha avanzado en el desarrollo e implementación de normas y procedimientos para mejorar estándares de calidad y oportunidad en la realización y evaluación de estudios clínicos desde la perspectiva de la agencia regulatoria, es necesario que también se revise y actualice la regulación que involucra a otros actores e instituciones participantes en la investigación clínica y contar con un marco regulatorio que se adapte a los desafíos que presentan las nuevas tecnologías en salud para garantizar la protección de los sujetos participantes y la calidad de las investigaciones.

### Contribución de los autores

Todos los autores concibieron el estudio original. FS planificó el diseño del manuscrito. LP recolectó los datos primarios, todos los autores analizaron los datos, todos los autores participaron en la escritura y edición del manuscrito. Todos los autores revisaron y aprobaron la versión final.

### Agradecimientos

Los autores agradecen al Grupo de Investigación Clínica, Dirección de Medicamentos y Productos Biológicos, Instituto Nacional de Vigilancia de Medicamentos y Alimentos (INVIMA) de Colombia.

### Declaración

Las opiniones expresadas en este manuscrito son responsabilidad del autor y no reflejan necesariamente los criterios ni la política de la *RPSP/PAJPH* y/o de la OPS.
